# Isolation and initial propagation of guinea pig adenovirus (GPAdV) in *Cavia porcellus* cell lines

**DOI:** 10.12688/f1000research.20135.2

**Published:** 2020-03-16

**Authors:** Adriana E. Kajon, Xiaoxin Li, Gabriel Gonzalez, Susan Core, Helga Hofmann-Sieber, Shuguang Leng

**Affiliations:** 1 Lovelace Respiratory Research Institute, Albuquerque, New Mexico, 87108, USA; 2 Research Center for Zoonosis Control, Hokkaido University, Sapporo, 001-0020, Japan; 3 Heinrich Pette Institute, Leibniz Institute for Experimental Virology, Hamburg, 20251, Germany

**Keywords:** Mastadenovirus, guinea pig, cell culture, PCR, phylogeny, nucleotide sequence

## Abstract

**Background: ** The lack of adequate *in vitro* systems to isolate and propagate guinea pig adenovirus (GPAdV), a prevalent cause of respiratory illness of varaible severity in laboratory guinea pig colonies worldwide, has precluded its formal characterization to allow for the development of comprehensive diagnostic assays, and for the execution of complex pathogenesis and basic virology studies.

**Methods:** Two strains of GPAdV were isolated in guinea pig ( *Cavia porcellus*) cell cultures from frozen archival infected animal tissue originated from colony outbreaks of pneumonia in Australia and the Czech Republic in 1996.

**Results:** Commercially available guinea pig cell lines from colorectal carcinoma (GPC-16), fetal fibroblast (104-C1) and lung fibroblast (JH4 C1), and the tracheal epithelial cell line GPTEC-T developed in this study were able to support viral infection and early propagation. Sufficient viral DNA was recovered from cell cultures to PCR-amplify and obtain sequence data for the complete hexon gene and partial DNA polymerase and penton base genes. Phylogenetic analysis for the three regions of the genome provided strong evidence confirming GPAdV as a unique species in the genus Mastadenovirus.

**Conclusions: **This study demonstrated the feasibility of propagating GPAdV in cultures of immortalized lines of GP cells of a variety of types, thus establishing a critical foundation for the development of a robust culture platform for virus stock production and titration. The generation and analysis of whole GPAdV genome sequences will provide additional data for a comprehensive description of the genetic organization of the viral genome and for a better assessment of genetic diversity between the two isolated strains.

## Introduction

Adenovirus (AdV)-associated pneumonia has been widely recognized as a cause of morbidity and occasional death in laboratory guinea pig (GP, *Cavia porcellus*) colonies worldwide since the early 1980s ( Brennecke *et al.*, 1983; Crippa *et al.*, 1997; Eckhoff *et al.*,1998; Feldman *et al.*, 1990; Finnie *et al.*, 1999; Naumann *et al.*, 1981; Shankar, 2008). However, knowledge about the alleged causative agent, guinea pig adenovirus (GPAdV), and the pathogenesis of the documented disease is still very limited. Published cases document infections of low morbidity and variable susceptibility, likely as a result of host age differences, immune competence and/or strain differences. The lack of adequate *in vitro* systems to isolate and propagate GPAdV has precluded its formal characterization to allow for the development of diagnostic assays and reagents, and for the execution of complex pathogenesis and basic virology studies. The only molecular data currently available for GPAdV in public databases is limited to a 234-nucleotide sequence of the hexon gene contributed by Pring-Akerblom and colleagues in 1997.

In this paper we report the development of a novel GP tracheal epithelial cell line, the feasibility of virus propagation of GPAdV in this and other commercially available GP cell lines, and the preliminary genetic characterization of two virus strains isolated from infected animal tissue originating from two different continents.

## Methods

### Sources of virus

GPAdV-infected lung tissue originating from a 1996 cluster of pneumonia cases in a laboratory GP (strain not specified) colony in Melbourne, Australia ( Finnie *et al.*, 1999) was obtained as a generous gift from the Veterinary Services Division, Institute of Medical and Veterinary Science to author AEK. Nasal scrapings from outbred Ibm:GOHI GPs (specific pathogen-free, Biological Research Laboratories Ltd., Füllinsdorf, Switzerland) experimentally infected with lung homogenates of GPs diagnosed with bronchopneumonia during a colony outbreak in Prague, Czech Republic in 1996 ( Butz *et al.*, 1999) were obtained as a gift from the Wolf laboratory at the Section of Comparative Medicine, Yale University School of Medicine to author AEK.

### Cell lines and culture conditions

GP ( *Cavia porcellus*) cell lines JH4 clone 1 (lung fibroblasts; RRID: CVCL_4299, catalog # CCL-158), 104-C1 (fetal fibroblasts; RRID: CVCL_2267, catalog # CRL-1405), and GPC-16 (colorectal adenocarcinoma; RRID: CVCL_3463, catalog # CCL-242) were purchased from ATCC and maintained and propagated as recommended by the vendor: JH4 clone 1 cells were grown in Hams’s F12K medium supplemented with 10% Fetal Bovine Serum (FBS); 104 C1 cells were grown in RPMI 1640 supplemented with 10% FBS; and GPC-16 cells were grown in Minimum Essential Medium (MEM) Eagle supplemented with 10% FBS. For infection, the FBS content was reduced to 2%. Cell monolayers were photographed in an Olympus CKX41 inverted microscope (Olympus Corporation) equipped with an Infinity 2 camera (Teledyne Lumenera).

### Source of GP tracheas

No live animals were involved in the study. We obtained the tracheas from two 2-month old male Hartley GPs that had been euthanized by intraperitoneal injection of a lethal dose of sodium pentobarbital after being used for laboratory animal technician training under Lovelace Respiratory Research Institute (LRRI) Institutional Animal Care and Use Committee (IACUC)-approved protocols. The tracheas were dissected at LRRI’s necropsy core and collected in 1X phosphate-buffered saline (PBS) for transportation to the Kajon laboratory in a refrigerated container within an hour.

### Immortalization of GP tracheal epithelial cells

The two tracheas were sliced longitudinally, the epithelial surface was scraped using a convex surgical scalpel, and the scraped material from both tracheas was pooled and collected into a 50 mL conical tube containing sterile wash medium [1X PBS and 2X penicillin/streptomycin (GIBCO/ThermoFisher Scientific)]. The cell suspension was centrifuged at 800 rpm (150 RCF) for five minutes at 4°C with no break. The supernatant was aspirated off and the pellet was resuspended in 10 mL of wash medium. This process was repeated twice. After the final wash, the cell pellet was resuspended in 12 mL of primary cell growth medium [50% DMEM and 50% Ham's F-12 supplemented with 15 mM HEPES (Invitrogen/ThermoFisher Scientific), 3.6 mM sodium bicarbonate (Sigma), 2 mM L-Glutamine (Invitrogen/ ThermoFisher Scientific), 5 µg/ml insulin (Lonza), 5 µg/ml transferrin (Lonza), 20 ng/ml cholera toxin (Lonza), 12.5 ng/ml epidermal growth factor (EGF, Lonza), 30 µg/mL Bovine Pituitary Extract (BPE, Lonza), 0.01 µM retinoic acid (Lonza), and 5% DMSO (Fluka 41639)]. In order to select against the fibroblast population present in the primary cell preparations, the culture medium contained no serum. A fraction of the cells were plated for propagation for two to three passages and the rest of the primary cell preparation was aliquoted into cryovials, frozen at -80°C and then moved for long term storage to liquid nitrogen.

For propagation in preparation for transfection, frozen cells at passage three were thawed and centrifuged at 800 rpm (150 RCF) for five minutes at 4°C with no break with 10 mL growth medium to remove DMSO. Cells were then resuspended in 10 mL of growth medium, plated onto a collagen-coated 96-well plate (Biocoat™ Matrigel™, catalog #354607, BD) and maintained at 37°C and 5% CO _2_. Twenty days after plating, monolayers were transfected with mammalian expression plasmid pRSVneoT, a generous gift of Dr. Jim Pippas (University of Pittsburgh) using PolyMag Transfection Reagent (OZ Biosciences USA, San Diego, CA), and Magnetofection™ protocols recommended by the manufacturer. Briefly, pRSVneoT DNA was diluted in serum free medium and mixed with PolyMag™ transfection reagent (catalog # PN30100) in various proportions in order to deliver 200 ng, 500 ng or 1 µg of plasmid DNA per well in 180 µl. Mixes were incubated for 20 minutes at room temperature; the transfection complexes were then added directly to cells, and the culture plate was placed on a 96-magnets plate (catalog # MF10096) for 20 minutes. The magnetic plate was then removed, and the cells were cultured overnight. The next day, the culture medium was replaced with fresh medium containing 0.4 mg/ml of G418. The transfected cells were kept in culture for four weeks and monitored for proliferation.

A single viable clone was recovered and further propagated to establish a cell line. The line was frozen at several stages of serial passaging (passages three, five and six) in liquid nitrogen for long term preservation. After five initial passages in nutrient rich medium, the line was adapted to grow in Eagle Minimum Essential Medium (MEM) supplemented with 8% New Born Calf Serum (NBCS), L-glutamine, HEPES 25mM, 7.5% Sodium Bicarbonate, and penicillin/streptomycin, without G418. For infection, the NBCS content was reduced to 2%.

### Karyotype analysis

Chromosomes were prepared, fixed, and stained following a modification of the protocol used by Costa *et al.* (2005). Briefly, cells at passage 10 were grown in a 75 cm ^2^ flask. When the cell confluence reached 80–90%, Colcemid™ ( *N*-methyl- *N*-deacetyl-colchicine, Sigma-Aldrich) was added to the cultures at a final concentration of 0.06 mg/ml to arrest cells in metaphase. Two hours later, cells were harvested by trypsin digestion and subsequently treated with 0.075 M KCl hypotonic solution for 30 minutes at 37°C and then fixed with methanol-acetic acid 3:1 at 4°C overnight. Air dried slides were stained with Giemsa. A total of 105 well-spread metaphases were examined by light microscopy under an oil immersion objective to count the number of chromosomes per metaphase.

### GPTEC-T cell line authentication

The GPTEC-T line at passage 25 was submitted to Leibniz Institute DSMZ, Braunschweig, Germany for authentication services. Cytochrome c oxidase subunit 1 (CO1) DNA bar coding ( Hebert *et al.*, 2003) was performed following institutional standard operating procedures based on the protocols described by Ivanova and colleagues ( Ivanova *et al.*, 2012). Detection of the SV40 T antigen coding sequence by PCR was carried out on total DNA extracted from cell suspensions using QIAamp DNA Micro Kit (catalog # 56304, QIAGEN, Valencia, CA) using primers F-SV-40 5’-GACTACAAGGATGACGATGACAAGCTC-3’ and R-SV-40 5’-CTCCACACAGGCATAGAGTGTCTG-3’. Cell lines from the DSMZ collection, HeLa (DSMZ ACC 057) and 293 (DSMZ ACC 305), and cell line 104C1 (ATCC CRL-1405) were used as negative controls. Cell line 293T (DSMZ ACC 635) was used as a positive control for the presence of the SV40 T antigen coding sequence.

### Indirect immunofluorescence

Cell monolayers on cover slips were fixed using 4% paraformaldehyde, followed by quenching using 10mM glycine and permeabilization with 0.5% Triton X-100. Cells were washed and blocked for one hour in 2% normal goat serum. Staining for epithelial cell marker ZO-1 was carried out with ZO-1 rabbit polyclonal antibody (RRID: AB_2533938, catalog # 61-7300, Zymed/ ThermoFisher Scientific) and Cyanine Cy™3-conjugated goat anti-rabbit polyclonal secondary antibody (RRID: AB_2338000, catalog # 111-165-003, Jackson ImmunoResearch;). Co-staining for SV40 T antigen was carried out with mouse monoclonal PAb416 (Abcam; RRID: AB_302561) and Cyanine Cy™5-conjugated goat anti-mouse polyclonal secondary antibody (RRID: AB_2338715, catalog # 115-175-205, Jackson ImmunoResearch). Nuclei were counterstained with Hoechst 33342 (Pierce/ ThermoFisher Scientific). Images were obtained at a 40X magnification in a Zeiss Axioskop epifluorescence microscope using Slide Book 6 digital microscopy software (Intelligent Imaging Innovations, Inc.).

### PCR detection of GPAdV DNA in infected materials

A modification of the PCR protocol developed by Pring-Åkerblom & colleagues (1997) was used to test the original materials obtained as sources of GPAdV, and to monitor virus propagation and serial passaging in cell culture. Lung homogenate supernatants, the nasal scrapings suspension and infected cell lysates were processed for total DNA extraction using QIAamp DNA Micro Kit (catalog #56304, QIAGEN, Valencia, CA). A total of 10 µl of QIAgen column-eluted DNA was added to each 40 µL reaction mixture containing 50 mM KCl, 10 mM Tris-HCl (pH 9.0), 1.5 mM MgCl _2_, 0.1% Triton X-100, 0.2 mM of each dNTP, 0.2 µM of each GPAdV-specific primer (GPAdV1: 5'-GCCAGGAGGCGGTAGAC-3' and GPAdV2: 5'-CCAAGACGCGATTGTCT-3'), and 1.25U of GoTaq® DNA polymerase (catalog # M3001, Promega Corporation, Madison, WI) following manufacturer-recommended thermal cycling conditions for GoTaq® DNA polymerase-mediated PCR (an initial step of denaturation at 95°C for two minutes, 35 to 50 cycles of denaturation at 95°C for 30 seconds, annealing at 60°C for 30 seconds, and extension at 72°C for 45 seconds; the final cycle had a prolonged extension time of five minutes). PCR products were analyzed by horizontal gel electrophoresis in 1% GeneMate LE agarose (catalog # E-3120-500, BioExpress/VWR, Radnor, PA) gels, prepared and run in 1X TBE buffer (0.09 M Tris-borate, 2 mM EDTA, pH 8.0). Gels were stained with ethidium bromide and visualized and photographed in a Gel-Doc imaging system (Bio-Rad, Hercules, CA). To control for the specificity of the reaction in the absence of positive controls, amplicons were submitted for Sanger sequencing with the amplification primers to Genewiz (South Plainfield, NJ).

### Amplification and sequencing of GPAdV hexon, penton base and DNA polymerase genes

The sequences of the complete hexon gene, partial DNA polymerase gene and partial penton base gene were determined for the preliminary characterization of virus isolates, and for confirmation of their identity as GPAdV. Degenerate primers spanning conserved regions based on analysis of available mammalian adenovirus sequences were designed to generate the initial amplicons. Degenerate primers were designed based on the alignment and analysis of a variety of known mastadenovirus hexon genes, upstream pVI genes, downstream protease genes, DNA pol genes and penton base genes. EcoRI or BamHI extension sites were incorporated to the 5’ end of these degenerate primers to facilitate cloning for subsequent sequencing. PCR products were gel-purified, digested with EcoRI and BamHI (Promega), and cloned into EcoRI-BamHI-digested and Calf Intestinal Alkaline Phosphatase (Promega Corporation, Madison, WI) -treated pUC19. Plasmid DNA was purified with GenElute ^TM^ Plasmid Miniprep Kit (catalog # PLN350-1KT Sigma) and sequenced with primers pUC19FW (5‘-TGCAAGGCGATTAAGTTGGG-3‘) and pUC19RV (5‘-CTTCCGGCTCGTATGTTGTG-3‘). PCR products obtained with other primers were sequenced directly using the specific amplification (or other appropriate) primers. Sanger sequencing services were contracted from Genewiz (South Plainfield, NJ). All primers used in this study were purchased from Integrated DNA Technologies, Inc. (Coralville, IA). A complete list of all primers used for the amplification and sequencing of the three regions of the GPAdV genome is provided in Table 1. PCR was carried out in a total volume of 50 µl with nuclease free water, PCR primers at a final concentration of 0.5 µM, 100–200 ng of total DNA, dNTP at a final concentration of 0.2 mM, 1 x iProof HF Buffer, 0.5 µl of High-Fidelity iProof DNA Polymerase (catalog # 1725300, Bio-Rad, Hercules, CA). Alternatively, for longer amplicons or difficult template regions PCR was also carried out using TaqSelect 2X Premix (catalog # 30041, Lucigen Corporation) containing dNTP, buffer and high-fidelity TaqSelect DNA Polymerase. PCR cycling conditions for product amplification varied with the primer pair and DNA polymerase (TaqSelect or iProof) but followed the corresponding manufacturers’ recommendations. The initial denaturation was carried out at 94°C for two minutes when using TaqSelect and at 98°C for 30 seconds when using iProof. When using TaqSelect, annealing temperatures were set at 2°C below the lowest calculated melting temperature (Tm). When using iProof, primers longer than 30 nt were annealed at 3°C above the lowest Tm for 30 seconds and primers 20 nt or shorter were annealed at a temperature equal to the lowest Tm for 30 seconds. The extension step was carried out at 72 °C for one minute/kb for TaqSelect, and 15 seconds/Kb for iProof. The initial denaturation step was followed by 35 cycles of [denaturation-annealing-extension] and an extra five minutes at 72°C for final extension and holding at 4°C.

**Table 1.** Primers used for the amplification and sequencing of the complete hexon, and partial penton base and DNA polymerase genes.

Targeted region of the GPAdV genome	Primer designation	Sequence
DNA polymerase (partial)	Pol FW 1	5’-TNMGNGGNMGNTGYTAYCC-3’
	Pol RV 1	5’-GTDGCRAANSHNCCRTABARNGMRTT-3’
	Pol internal FW1	5’GTNTWYGAYATHTGYGGHATGTAYGC-3’
	Pol internal RV1	5’-CCANCCBCDRTTRTGNARNGTRA-3’
	GpPolFW2	5’-GTACACGTACACGGGATGGG-3’
	GpPolRV2	5’-CGGTGCACAAGGGACACTG-3’
	GpPolFW3	5’-GCACGAAGCGCTCGTAACTG-3’
	GpPolRV3	5’-GGCACAACATCAGCGGGTTC-3’
	GpPolRV4	5’GCGGGTTCGACGAGATCGTG-3’
Penton base (partial)	GPpenton FW1BamHI	5’-GCGGATCCAAYWWSCARAACRACCAC-3’
	GPpenton RV1EcoRI	5’-GCGGAATTCAGRTAYMARCTKCGGTA-3’
	GPpenton Fw2BamHI	5’-ATGGATCCGAYRASCGBTCSCGBTGGGG-3’
	GPpenton RV2EcoRI	5’-GCGGAATTCTTRTASACRTARGGRCA-3’
	GPpenton FW1	5’-AATTCCGCAGCGCCCTGAAC-3’
	GPpenton FW2	5’-GCGCCCTGAACACCAACC-3’
	GPpenton RV1	5’-GTAGGCCGTGAAGGTCG-3’
	GPpenton RV2	5’-CAGGGCGGGAATATTGC-3’
Hexon (complete coding sequence)	GPhex FW BamHI	5’-TAGGATCCAACACGGGTCTGCGCTATCG-3’
	GPhex3’end RV EcoRI	5’-CGGAATTCTTAKGTGGTGGCGTTNCCGGC-3’
	GPAdV hex Fel FW ^¥^	5’-ATGCACATCGCCGGCCAG-3’
	GPhex5’endRvEcoRI	5’-CGGAATTC-ACAAACCGCAGTTGGAGTC-3’
	GPAdV hex Fel RV ^¥^	5’-AAGBWCCACTCRTARGTGTA-3’
	GPpVIFw1BamHI	5’-ATGGATCCGGCAHSAGYSARMTGMACGG-3’
	GPpVIFw2BamHI	5’-ATGGATCCTGGRGYARTNTVTGGAG-3’
	GPpVIFw	5’-AGTAGAAAGCGGTACAGAGG-3’
	GPprotease RV1EcoRI	5’-GCGGAATTCAANGGRTCRAASADGTA-3’
	GPprotease RV2EcoRI	5’-ATGAATTCAAVAGHCCRCASGCKGC-3’
	GPprotease RV3	5’-AGGAAAGGTGCTGTCGAACG-3’
	GPprotease RV4	5’-CGTAGGGATCGAACATGTAG-3’
	GPhexFW1	*5’*-TCGGGAGGACACTCAGTATG- *3’*
	GPhexFW2	5’-CGCAACACCTACGCTTAC-3’
	GPhexFW3	5’-GACTCCCGACACGCATCTG-
	GPhexFW4	5’-GCTGCTGTTAGACAACC-3’
	GPhexFW5	5’-AACACGGGTCTGCGCTATC-3’
	GPhexFW6	5’-GTCCTTCCGCAAAGACG-3’
	GPhexRV1	5’-AGGTTTCCGCTGCTGTTG-3’
	GPhexRV2	5’-ACGACACGGAATGGAGATG-3’
	GPhexRV3	5’-TTAGCGGGTACGGCCAGTTG-3’
	GPhexRV4	5’-CATGGCCTGAACGTTGC-3’
	GPAdV1 ^^^	5’-GCCAGGAGGCGGTAGAC-3’
	GPAdV2 ^^^	5’-CCAAGACGCGATTGTCTC-3’
	GPhexon5’end	5’-ATGCCACAGTGGTCGTACATGCACATATC-3’

**¥** Feldman *et al.*, 2001; **^** Pring-Akerblom *et al.*, 1997

### Assembly and phylogenetic analysis of hexon, DNA polymerase and penton base sequences

Sequence reads were edited in EditSeq 15 (Lasergene® Molecular Biology Suite, DNASTAR) to remove vector sequences in and poor quality sections. Contigs were assembled in Seqman Pro 15 (Lasergene® Molecular Biology Suite, DNASTAR). Sequences for the complete hexon gene and partial DNA polymerase and penton base genes were deposited in GenBank under accession numbers MK787233- MK787238 (see *Underlying data*).

Multiple sequence alignments (MSAs) were prepared with partial or complete sequences of GPAdV and homologous sequences in other mastadenovirus species of corresponding to loci encoding the DNA polymerase, penton base and hexon. The fowl adenovirus 8b strain 764 ( KT862811 FAdV-D) was used to root the adenovirus species isolated from mammals. Adenoviral species isolated from mammals considered for these comparisons were: AB026117 porcine adenovirus 3 (PAdV-3), KY306667 deer adenovirus B (Odocoileus hemionus adenovirus, OdAdV-B), AF252854 bovine adenovirus 2 (BAdV-2), AF030154 bovine adenovirus 3 (BAdV-3), AC_000003 canine adenovirus 1 (CAdV-1), X73487 human adenovirus A12 (HAdV-A12), AY594255 human adenovirus B7 (HAdV-B7), AC_000008 human adenovirus C5 (HAdV-C5), KT862547 human adenovirus D8 (HAdV-D8), AP014853 human adenovirus E4 (HAdV-E4), KU162869 human adenovirus F40 (HAdV-F40), DQ923122 human adenovirus G52 (HAdV-G52), AC_000012 murine adenovirus 1 (MAdV-1), HM049560 murine adenovirus 2 (MAdV-2), EU835513 murine adenovirus 3 (MAdV-3), KP329564 simian adenovirus 16 (SAdV-16), FJ025929 simian adenovirus 47 (SAdV-47), HQ241820 simian adenovirus 50 (SAdV-50), KX505867 simian adenovirus (SAdV WIV19), HQ913600 titi monkey adenovirus (TMAdV), KT013209 cynomolgus Adenovirus (CynAdV), MF198459 rhesus adenovirus 65 (SAdV-65), AF258784 tree shrew adenovirus 1 (TsAdV-1), JN252129 bat adenovirus 2 (BtAdV-2), KT698853 bat adenovirus WIV9 (BtAdV-WIV9), and JN418926 equine adenovirus 1 (EAdV-1).

MSAs were built with MAFFT v7.429 ( Katoh & Standley, 2016) using the FFT-NS-i algorithm. The phylogenetic relation of both GPAdV were explored with Bayesian and maximum likelihood methods with MrBayes v3.2.6 ( Ronquist *et al.*, 2012) and RAxML v8.2.10 ( Stamatakis, 2014), respectively. In both cases, the general time-reversible substitution model with a gamma-modeled heterogeneity among sites and allowing for invariant sites (GTR+I+G), was used for the phylogenetic inference. The substitution model was chosen using the best Aikaike information criterion corrected (AICc) inferred with jmodeltest 2 v0.1.10 ( Darriba *et al.*, 2012). MrBayes was executed with five million states per chain to assure convergence. The support in branches for the topology inferred by RAxML was calculated with bootstrap on 5000 repetitions.

## Results

### Generation and characterization of GPTEC-T, an immortalized line of GP tracheal epithelial cells

A novel GP epithelial cell line was generated by transfection of primary tracheal epithelial cells from a Hartley male GP with plasmid pRSVneoT as described in the *Methods* section. The new cell line designated GPTEC-T to denote its origin and features.

**Figure 1.**
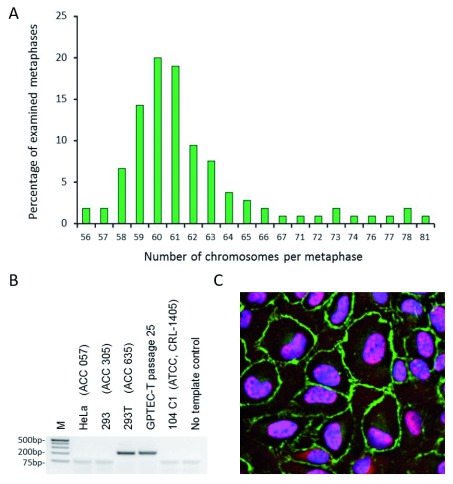
Characterization of guinea pig tracheal epithelial cell line GPTEC-T. **A**. Chromosome numbers in metaphases of GPTEC-T cells at passage 23. A total of 105 metaphases were examined by light microscopy. **B**. Detection of T antigen coding sequence by PCR for quality control at DSMZ, Germany. Total DNA was extracted from GPTEC-T and various other cell lines included in the test as controls, and used as a template. M: Molecular marker Generuler 1kB plus (Thermo Fisher Scientific). **C**. Indirect immunofluorescence staining of GPTEC-T cells for ZO-1 and SV40 T antigen. Cells were stained with primary antibodies anti ZO-1 and anti SV40 T antigen and appropriate secondary antibodies as described in the Methods section. Nuclei were stained with Hoechst. Images were acquired with a Zeiss Axioskop epifluorescence microscope at 40X magnification. Merged image of ZO-1 staining at the plasma membrane (green) and nuclear staining for T antigen (red) and Hoechst (blue).

GPTEC-T cells at passage 23 were karyotyped at LRRI. While the diploid number of chromosomes for *Cavia porcellus* is 64, data from the examination of 105 GPTEC-T metaphases demonstrated aneuploidy, showing a distribution of 56 - 81 chromosomes with 60 and 61 chromosomes found in 20 and 19% of the metaphases ( Figure 1A).

The GPTEC-T line was further authenticated by CO1 DNA bar coding as of *Cavia porcellus* origin at Leibniz Institute DSMZ and confirmed to encode SV40 T antigen coding region by PCR ( Figure 1B). As shown in Figure 1C, examination of GPTEC-T cells by indirect immunofluorescence demonstrated that the line expresses epithelial marker ZO-I and, as expected, SV40 T antigen.

### Recovery of GPAdV from infected cell cultures

**Figure 2.**
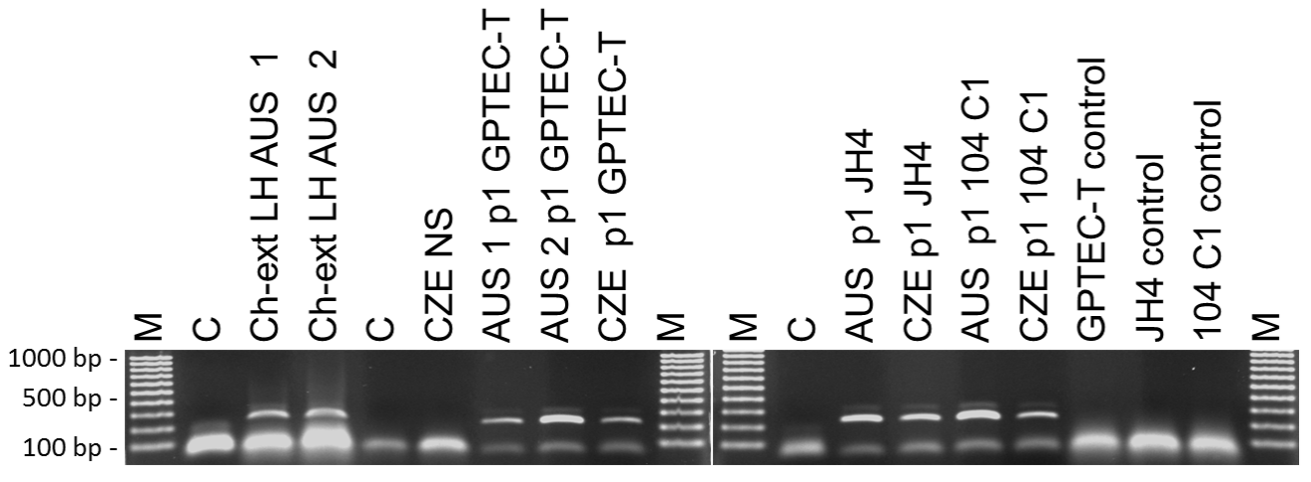
Detection of GPAdV DNA in infected tissues and cell cultures. Total DNA was extracted from GP lung homogenate, nasal scrapings or cell culture aliquots with Qiagen DNA micro kit (QIAGEN, Valencia, CA) and used as a template for PCR amplification of a portion of the hexon gene using the primers and protocol developed by Pring-Akerblom *et al.* (1997). **Ch-Ext. LH**: chloroform-extracted lung homogenate supernatant; **AUS 1 and AUS 2**: designations for two different Australian lung tissue samples; **CZE NS**: nasal scraping suspension from GPs experimentally infected with Czech Republic strain; **C**: negative control; **M**. EZ Load™ 100 bp Molecular Ruler (Bio-Rad, Hercules, CA); **p1**: first passage in culture from original sample.

**Figure 3.**
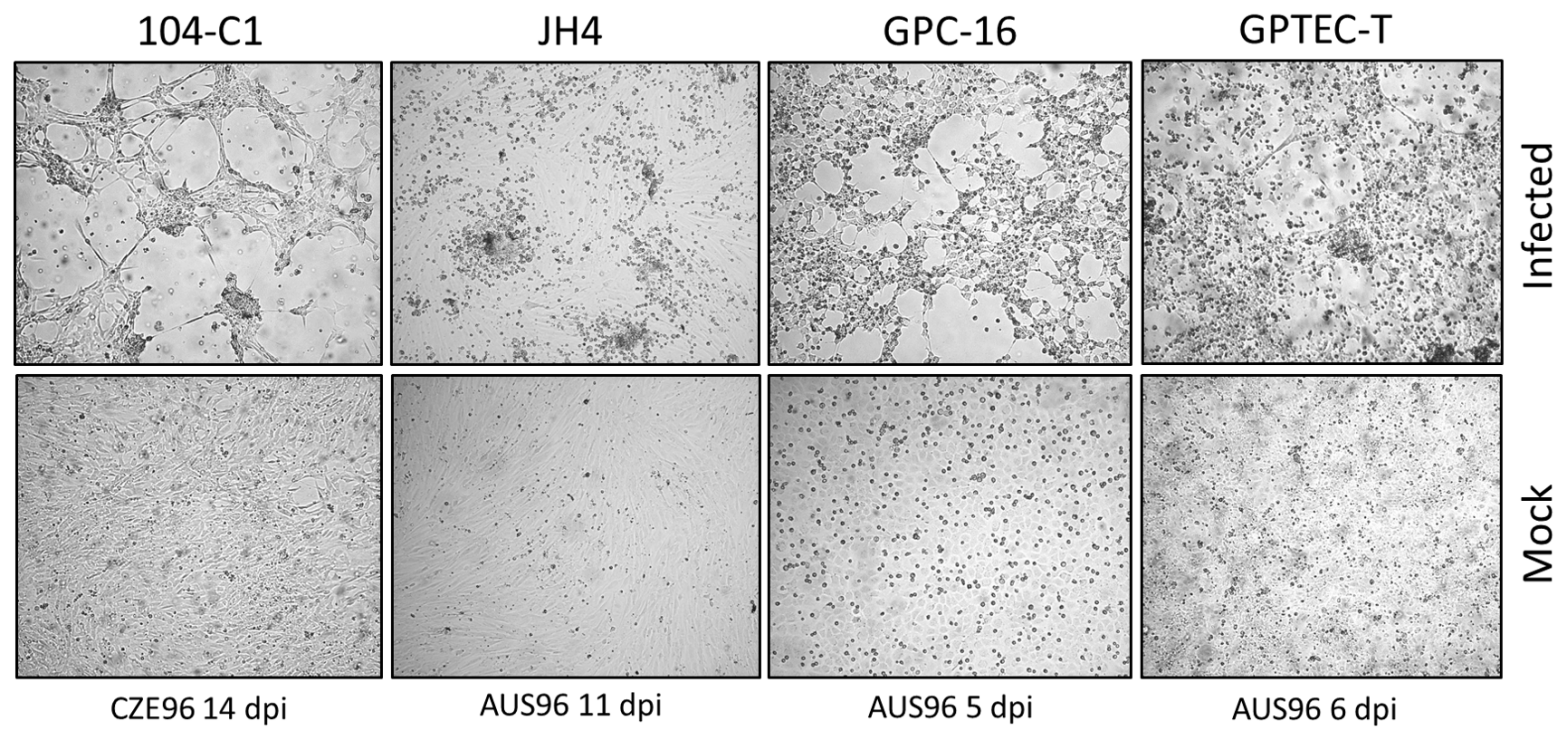
Representative cytopathic effect observed in cell cultures infected with PCR-positive passage three of strains AUS96 and CZE96. Infected cultures and uninfected controls were examined daily for the occurrence of cytopathic effect. Photographs were taken at various days post infection (dpi) as indicated at 20X magnification in an Olympus CKX41 inverted microscope (Olympus Corporation) equipped with an Infinity 2 camera (Teledyne Lumenera).

GPTECT-T, GPC-16, JH4 and 104-C1 cell monolayers in 24-well plates were infected with 50 µl of lung homogenate supernatant, or 50 µl of nasal scraping and monitored for the development of cytopathic effect (CPE) for 7–14 days. PCR screening of original specimens and their passages in GP cell lines yielded the expected 281 bp fragment for all tested samples ( Figure 2). The identity of the amplified products was verified by Sanger sequencing. Representative amplicon sequences were deposited in GenBank under accession numbers MN250852 and MN250853. The original nasal scraping sample did not test positive by PCR, but passage one in culture did, demonstrating the presence of infectious virus in the original material. Amplicon sequences were 98–100% identical to the sequence submitted to GenBank by Pring-Åkerblom and colleagues under accession number X95630 ( *Guinea pig adenovirus hexon)*. CPE of variable magnitude was observable in monolayers infected with both sources of virus but not in the controls. The isolated strains were designated AUS96 and CZE96. In all tested cell lines, CPE was initially focal but increased in magnitude in passages two and three. Representative images from cell monolayers infected with PCR-positive passage three of AUS96 or CZE96 in the corresponding lines are shown in Figure 3.

### Preliminary genetic characterization of isolated virus strains

The complete hexon gene sequence and partial sequences for the penton base gene and DNA polymerase gene were successfully determined for both virus strains using our strategy of an initial round of amplification using degenerate primers followed by primer walking to obtain readings from both strands. We obtained partial sequence data for 789 bases and 801 bases of the DNA polymerase gene, and partial sequence data for 473 bases and 549 bases of the penton base gene for strains AUS96 and CZE96, respectively. Sequences were deposited in GenBank under accession numbers MK787233- MK787238.

While the partial sequences for the DNA polymerase gene were identical between both isolated strains, a few nucleotide differences were found between strains AUS96 and CZE96 in the hexon gene and partial penton base gene sequences (available as *Underlying data*; Kajon *et al.*, 2019).

**Figure 4.**
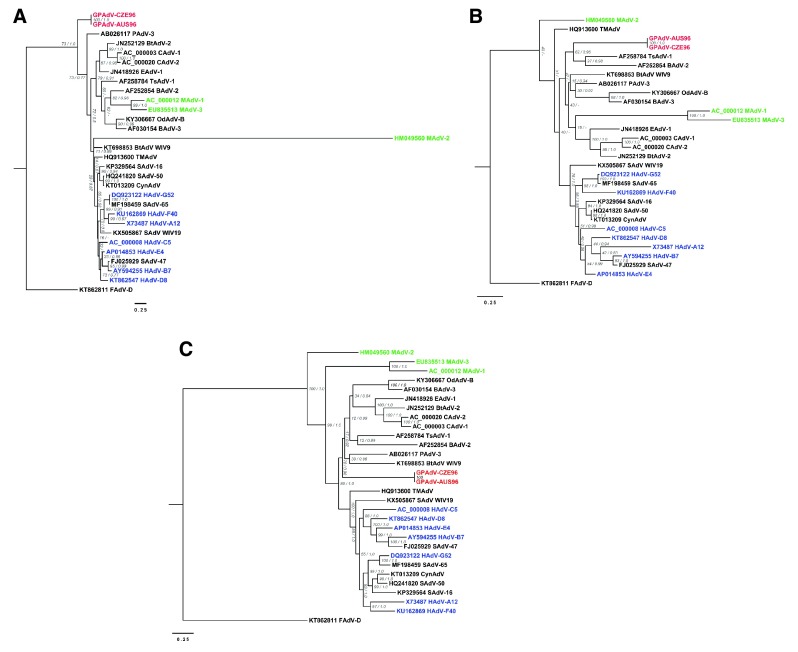
Phylogenetic relationships of GPAdV strains AUS96 and CZE96 and other mastadenoviruses. Sequences obtained for the DNA polymerase (partial), penton base (partial) and hexon genes for the two isolated GPAdV strains were aligned with sequences for the corresponding regions of the genome of twenty seven (27) selected mastadenoviruses in MAFFT using the FFT-NS-i algorithm. The phylogenetic relationships of both GPAdV strains were explored with Bayesian and maximum likelihood methods with MrBayes and RAxML, respectively. The maximum likelihood-inferred phylogenetic trees were rooted with the sequence of fowl adenovirus 8b strain 764 ( KT862811 FAdV-D). Branches are annotated with the bootstrap support (RAxML) and the posterior probability (MrBayes). The absence of posterior probability in the Bayesian topology is marked with a dash (-). Tip names from adenoviral species isolated in human, murine, and guinea pig hosts, are colored in blue, green and red, respectively. The scale represents 0.25 nucleotide mutations per site. **A**. Phylogenies inferred using the partial nucleotide sequence of the DNA polymerase gene. **B**. Phylogenies inferred using the partial nucleotide sequence of the penton base gene. **C**. Phylogenies inferred using the nucleotide sequence of the complete hexon gene.

**Figure 5.**
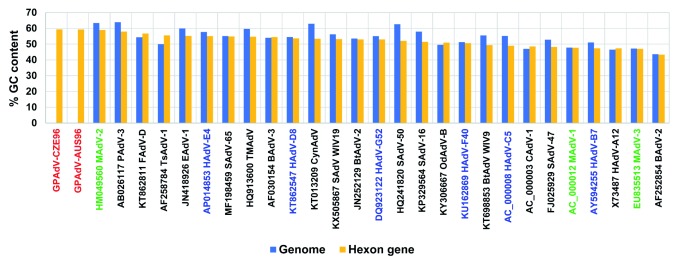
Comparison of the GC content of the GPAdV hexon gene to those encoded by other mastadenovirus species. The percentage of cytosine and guanine (%GC) per sequence was calculated for the hexon gene sequences (orange) and for available complete genomic sequences (blue) as context. The vertical axis represents the % GC and the compared mastadenovirus sequences are shown in the horizontal axis on descending order according to the %GC in the hexon gene.

Our preliminary phylogeny reconstructions for GPAdV based on partial nucleotide sequences for the DNA polymerase, and penton base genes and on complete hexon gene sequences are presented in Figure 4 A–C. The three trees show that the virus is a novel mastadenovirus genetically distinct from other species in the genus. A relatively high GC content of 59.30% and 59.34% was found in the hexon gene sequences of strains AUS96 and CZE96, respectively ( Kajon *et al.*, 2019). A comparison of GC contents among the examined mastadenoviruses is shown in Figure 5.

## Discussion

The lack of suitable culture systems for the isolation and propagation of GPAdV has been a major impediment in the development of this virus as a potential model system for the study of adenoviral pathogenesis. This study established the feasibility of propagating, albeit at an apparent low efficiency (based on the observation of modest cytopathic effect), GPAdV in cultures of immortalized lines of GP cells of a variety of types. This important matter warrants further investigation and efforts to establish a robust culture platform for virus stock production and titration. In addition, the availability of a variety GP cell lines and molecular assays to support and monitor viral replication will be critical for the study of virus-host interactions *in vitro.*

Our initial culture efforts recovered enough viral DNA to generate sequence data for the complete hexon gene and partial sequence data for the DNA polymerase and penton base coding regions for both isolated strains, AUS96 and CZE96.

Our sequence data and analysis complement the findings of Pring-Åkerblom and colleagues’ (1997) and Feldman and colleagues’ (2001) in providing evidence that GPAdV is a unique species in the genus Mastadenovirus. Interestingly, our data also show the two strains isolated from infected GPs in geographically distant locations to be surprisingly closely related.

The topology of the tree generated in this study for the complete hexon gene is similar to the one reported by Feldman and colleagues (2001) using a 534 amino acid sequence inferred from a 5’ partial 1603 nucleotide sequence of the hexon gene. The original source of total DNA used as a template for PCR amplification by these investigators was lung tissue obtained from two cases of fatal pneumonia documented in the United States in 1997. Unfortunately, this sequence was never submitted to GenBank and could not be incorporated into our alignment for comparison.

The generation and analysis of whole genome sequences (WGS) will provide additional data for a comprehensive description of the genetic organization of the GPAdV genome and for a better assessment of genetic diversity between the two isolated strains. The high GC content identified for the hexon gene relative to that reported for other mammalian adenoviruses is intriguing and highlights the need for further in-depth analysis once the WGSs are available for these and other GPAdV strains. The GC content has been shown to vary widely among AdV genomes and to correlate with genome size and CpG representation ( Davison *et al.*, 2003; Tan *et al.*, 2017).

## Data availability

### Underlying data

GPAdV strain AUS96 complete hexon gene sequence on GenBank, Accession number MK787233

GPAdV strain CZE96 complete hexon gene sequence on GenBank, Accession number MK787234

GPAdV strain AUS96 partial DNA polymerase gene sequence on GenBank, Accession number MK787235

GPAdV strain CZE96 partial DNA polymerase gene sequence on GenBank, Accession number MK787236

GPAdV strain AUS96 partial penton base gene sequence on GenBank, Accession number MK787237

GPAdV strain CZE96 partial penton base gene sequence on GenBank, Accession number MK787238

GPAdV strain AUS96 partial hexon gene amplicon sequence on GenBank, Accession number MN250852

GPAdV strain CZE96 partial hexon gene amplicon sequence on GenBank, Accession number MN250853

Zenodo: Isolation and Initial propagation of guinea pig adenovirus (GPAdV) in Cavia porcellus cell lines. http://www.doi.org/10.5281/zenodo.3699878 ( Kajon *et al.*, 2019)

This project contains the following underlying data:

- 104C1p48 CZE 14dpi.jpg (raw, unedited image showing CPE in a CZE96-infected 104-C1 cell culture from Figure 3)
- 104C1p48 uninfected control 14dpi.jpg (raw, unedited image showing an uninfected 104-C1 cell culture control monolayer from Figure 3)
- GPC-16 AUS96 5dpi.jpg (raw, unedited image showing CPE in an AUS96-infected GPC-16 cell culture from Figure 3)
- GPC-16 p57 uninfected control 5dpi.jpg (raw, unedited image showing an uninfected GPC-16 cell culture control monolayer from Figure 3)
- GPTEC-T AUS96 6 dpi.jpg (raw, unedited image showing CPE in an AUS96-infected GPTEC-T cell culture from Figure 3)
- GPTEC-T p9(12) uninfected control 6 dpi.jpg (raw, unedited image showing an uninfected GPTEC-T cell culture control monolayer from Figure 3)
- JH4 p42 Aus 96 11 dpi.jpg (raw, unedited image showing CPE in an AUS96-infected JH4 cell culture from Figure 3)
- JH4 p42 control 11 dpi.jpg (raw, unedited image showing an uninfected JH4 cell culture control from Figure 3)
- GPTEC-T IF 40X Air Dapi.tif (raw, unedited immunofluorescence image showing Hoescht staining only)
- GPTEC-T IF 40X Air MERGED ZO-1 T antigen Dapi.tif (raw, unedited immunofluorescence image showing merged DAPI - ZO-1 and T antigen staining)
- GPTEC-T IF 40X Air T antigen.tif (raw, unedited immunofluorescence image showing T antigen staining only)
- Shuguang metaphase analysis.xls (raw karyotype counts underlying karyotype analysis)
- smcgrath 2008-02-06 PCR screening.jpg (raw, unedited gel image from Figure 2)
- Wilkens(E1)_170820181 GPTEC-T cell line authentication.pdf (results of DNA bar coding carried out by Leibniz Institute DSMZ)
- DSMZ SV40 T antigen PCR assay (GPTEC-T cell line testing for SV40 T antigen at DSMZ, original unedited gel obtained from DSMZ with the report)
- GC Content values.xls (raw data used in the generation of Figure 5)

Data are available under the terms of the Creative Commons Attribution 4.0 International license (CC-BY 4.0).
