# Supplementary material for: Isolation and initial propagation of guinea pig adenovirus (GPAdV) in Cavia porcellus cell lines
Source: F1000Res. 2020 Mar 16;8:1597. Originally published 2019 Sep 5. [Version 2] doi: 10.12688/f1000research.20135.2 (PMC7096189; doi:10.12688/f1000research.20135.2)
Supplement: Peer review history

# Peer review: Reviewer response for version 2

**DOI:** 10.5256/f1000research.25109.r61364

**Published:** 2020-03-23

**Authors:** Ana Paula Ravazzolo

**Affiliations:** 1 Faculdade de Veterinária, Laboratório de Imunologia e Biologia Molecular, Federal University of Rio Grande do Sul (UFRGS), Porto Alegre, Brazil

The authors have performed the majority of the suggested modifications. I have no further comments.

I confirm that I have read this submission and believe that I have an appropriate level of expertise to confirm that it is of an acceptable scientific standard.

# Peer review: Reviewer response for version 1

**DOI:** 10.5256/f1000research.22110.r58978

**Published:** 2020-02-19

**Authors:** Ana Paula Ravazzolo

**Affiliations:** 1 Faculdade de Veterinária, Laboratório de Imunologia e Biologia Molecular, Federal University of Rio Grande do Sul (UFRGS), Porto Alegre, Brazil

In this manuscript, the authors described the generation of a tracheal epithelial guinea pig cell line, as well as the isolation and molecular characterization of two strains of adenovirus from *Cavia porcellus *(GPAdV). Virus propagation and partial nucleotide sequences are important findings, leading to the establishment of methods to better characterize GPAdVs.

 Some minor points:

Title: Why using “initial propagation”? Why not “propagation” only?

Keywords: Adding “phylogeny”, “nucleotide sequence” or “molecular characterization” could be more informative.

Methods:

 In “Source of GP tracheas”, “…for transportation to the Kajon laboratory” do not specify the technical approach, like time and temperature until isolation and propagation of the cells.

 In “Immortalization of GP tracheal epithelial cells”, the sterile wash medium contains “2% penicillin/streptomycin”. Does it means 2 g of each one/100 mL of PBS? Alternatively, is it 2 times concentrated (2x)? The centrifugation will be better described in *g* than rpm. Why there are some additives to the culture medium (e.g. insulin, transferrin, etc.)? I suppose that they are used to select epithelial cells. If this is the case, some reference could be included.

 In PCR detection, it should be considered that positive controls are not mandatory to exclude false positives (as mentioned “To control for the specificity of the reaction in the absence of positive controls,…”)

Results:

 Recovery of the virus in different cell lines seems to be similar for all of those evaluated although pictures showed at Fig. 3 correspond to different time lapses. Could we consider or speculate about more permissive cell lines?

 Legends of Figures: Fig 4 presents a misspelling – “twety” for twenty; in Fig. 5 blue is designed for both, virus genome and hexon gene.

Discussion:

 It is not clear why authors mentioned “apparent low efficiency” of virus propagation. This speculation was based on PCR? In comparison with other adenovirus? I suggest citing the reference or the reason to this statement.

 In my opinion, there are some aspects to explore using the data obtained: differences of virus propagation among the cell lines, phylogeny, PCR positive result after virus propagation (nasal scraps) and comparisons with other described adenoviruses. One point that should be addressed is some low values of bootstrap and posterior probability at Fig. 4 to justify the conclusion “strong evidence confirming GPAdV as a unique species in the genus Mastadenovirus”.

I confirm that I have read this submission and believe that I have an appropriate level of expertise to confirm that it is of an acceptable scientific standard, however I have significant reservations, as outlined above.

# Response

**Published:** 2020-03-06

**Authors:** Adriana Kajon

**Affiliations:** Lovelace Respiratory Research Institute, USA

We would like to thank Dr. Ravazzolo for her comments on our manuscript. We have considered her suggestions and have introduced several edits as described below:

 -In this manuscript, the authors described the generation of a tracheal epithelial guinea pig cell line, as well as the isolation and molecular characterization of two strains of adenovirus from Cavia porcellus (GPAdV). Virus propagation and partial nucleotide sequences are important findings, leading to the establishment of methods to better characterize GPAdVs.

 Some minor points:

Title: Why using “initial propagation”? Why not “propagation” only?

 Response:

*We have introduced the term “initial” referring to the propagation of GPAdV in cell culture to denote that these are early efforts towards the development of a robust system that can host efficient viral replication for stock production and titration and to. Reflect our awareness that our cell culture systems may change dramatically in the future as we develop and test new GP cell lines or implement the use of certain inhibitors (such Ruxolitinib, a Jak 1 and Jak 2 signaling pathway inhibitor) that decrease levels of inflammatory cytokines potentially making the system more permissive to infection.*

-Keywords: Adding “phylogeny”, “nucleotide sequence” or “molecular characterization” could be more informative.

 Response:

*We have added “phylogeny” and “nucleotide sequence” to the original keyword list in the revised version of our manuscript.*

Methods:

 -In “Source of GP tracheas”, “…for transportation to the Kajon laboratory” do not specify the technical approach, like time and temperature until isolation and propagation of the cells.

 Response:

*The text was edited to clarify this aspect of the protocol and read **“ The tracheas were dissected at LRRI’s necropsy core and collected in 1X phosphate-buffered saline (PBS) for transportation to the Kajon laboratory in a refrigerated container within an hour”***

 -In “Immortalization of GP tracheal epithelial cells”, the sterile wash medium contains “2% penicillin/streptomycin”. Does it means 2 g of each one/100 mL of PBS? Alternatively, is it 2 times concentrated (2x)?

 Response:

*Dr. Ravvazolo is absolutely right. This was an unnoticed typo. We have corrected the text to read **“2X penicillin/streptomycin”**.*

 -The centrifugation will be better described in g than rpm.

 Response:

*The text in the Methods section was edited to read **“800 rpm (150 RCF)…”***

 -Why there are some additives to the culture medium (e.g. insulin, transferrin, etc.)? I suppose that they are used to select epithelial cells. If this is the case, some reference could be included.

 Response:

*These are standard protocols and medium formulations for primary cell culture so we have not provided any specific references. We did however edit the text to read **“primary cell growth medium”.***

 -In PCR detection, it should be considered that positive controls are not mandatory to exclude false positives (as mentioned “To control for the specificity of the reaction in the absence of positive controls,…”)

 Response:

*As written in the text, we DID NOT include positive controls. What we did was to submit amplicons for Sanger sequencing.*

Results:

 -Recovery of the virus in different cell lines seems to be similar for all of those evaluated although pictures showed at Fig. 3 correspond to different time lapses. Could we consider or speculate about more permissive cell lines?

 Response:

*We do not feel comfortable interpreting these results until we have a standardized system to titer input virus and monitor viral replication in culture.*

 -Legends of Figures: Fig 4 presents a misspelling – “twety” for twenty; in Fig. 5 blue is designed for both virus genome and hexon gene.

 Response:

*These corrections were incorporated into the revised version of the manuscript.*

Discussion:

 -It is not clear why authors mentioned “apparent low efficiency” of virus propagation. This speculation was based on PCR? In comparison with other adenovirus? I suggest citing the reference or the reason to this statement.

 Response

*We do not have a reference to provide for this observation and we do not have quantitative PCR in place yet. Our statement is based on the observation of very limited cytopathic effect in subsequent passages. We have edited the text to address this issue and read **“This study established the feasibility of propagating, albeit at an apparent low efficiency (based on the observation of modest cytopathic effect),…”***

 -In my opinion, there are some aspects to explore using the data obtained: differences of virus propagation among the cell lines, phylogeny, PCR positive result after virus propagation (nasal scraps) and comparisons with other described adenoviruses. One point that should be addressed is some low values of bootstrap and posterior probability at Fig. 4 to justify the conclusion “strong evidence confirming GPAdV as a unique species in the genus Mastadenovirus”.

 Response:

*We edited the text eliminating the word “strong" to read **“… in providing evidence confirming GPAdV as a unique species in the genus Mastadenovirus”.***

# Peer review: Reviewer response for version 1

**DOI:** 10.5256/f1000research.22110.r53516

**Published:** 2019-09-18

**Authors:** Márton Z. Vidovszky

**Affiliations:** 1 Institute for Veterinary Medical Research, Centre for Agricultural Research, Budapest, Hungary

This manuscript describes the isolation of two strains of guinea pig adenoviruses and their successful propagation on a newly developed and on three commercially available guinea pig (GP) cell lines. The manuscript is very relevant, as every successful, novel virus isolation is very important for adenovirology. The manuscript also discusses the partial phylogenetic comparison of adenoviral sequences with the examined AdV strains (full hexon gene and partial polymerase and penton base sequence parts).

  

- The Introduction is thorough enough and dense in a good meaning. It only deals with guinea pig AdVs, but all the known publications are well collected.
- The Methods section is a bit too detailed and long compared to the other parts of the manuscript. Sometimes the process is already published somewhere, and the manuscript refers to it correctly but also details shortly. This is unnecessary in some cases in my opinion.
- The process of the primary cell line production and its immortalization is very well written. It is easy to follow the process and very interesting.
- The successful isolation and propagation of the two GPAdVs are a very nice job and well written. The pictures of CPEs on different cell lines represent the changes correctly and well visualize this part of the research. The surprising parts of the adenoviral PCR detection in the homogenates and infected cell cultures are that the original nasal scraping sample was PCR negative for AdV, while the cell culture infected by this material showed CPE and it was PCR positive. I would be curious of the opinion of the authors, how this can be possible, i.e., that the PCR reaction is not enough sensitive for detection, but the material is suitable for the usually less successful virus isolation.
- It is a great result that the virus isolation attempts were successful with the GPAdV positive materials. It is more pity that the very interesting full genome sequence of these strains are not available. In the case of isolated viruses, it is only a small step by NGS. Because of the lack of full genome sequences of the examined AdVs, Figure 5. loses its relevancy. This makes some data incomparable in the figure.
- The phylogenetic calculations are very interesting, however, the one, based on the full hexon gene, seems to show a bit different topology than the other two. It is surprising, that among mastadenoviruses, not the rodent AdVs are the most ancient ones as usual, and they are not monophyletic with the GPAdVs.
- Summarizing, this is a well-written manuscript with very relevant and precious results.

 

**Minor notes:**

  

- There are a couple of not disturbing misspellings or inadvertent mistakes. The only one, which can be confusing is in the legend of Figure 5. The labeling is correct on the figure, but in the legend, both columns are labeled with blue instead of orange and blue. Other mistakes:
- Misspelling in the abstract/background: “variable” instead of “variable”.
- Twice, the word “resuspended” was misspelled to “resuspened” (Methods/Immortalization, 1 ^st^ paragraph). Also, here in the last sentence the word “aliquoted” was misspelled to “alliquoted”.
- In most of the times, it was used correctly, but several times the “u” character was used instead of “µ” e.g.: twice in Methods/Immortalization, 2 ^nd^ paragraph. Also, here a space is missing in one of the cases, between the number and the unit (1ug), correctly (1 µg). The lack of space mistake between the amount and the unit occurs several times in the manuscript. I won't detail all of them here. Please check them. Also, a dot is unnecessary in the middle of a sentence of this paragraph, after a catalog nr: (catalog # MF10096).
- A comma should be used in the listing instead of “and” at Methods/GPTEC-T cell line authentication, after (DSMZ ACC 057).
- The shortening of fowl adenovirus D was used incorrectly. The letter “a” of adenovirus is in lower case font. It should be a capital “A”. Everywhere else it was used correctly. (Methods/Assembly, 1 ^st^ paragraph: “FadV-D” was written instead of “FAdV-D”).
- Once, the word “adenovirus” was written also with a capital incorrectly in the case of cynomolgus adenovirus.
- A “3’” end is missing for primer GPhexFW3 in Table 1.
- The word “cytopathic” was misspelled to “cythopathyc” (legend of Figure 3.).
- The word “twenty” was misspelled to “twety” (legend of Figure 4.). Also, here the word “absence” was misspelled to “absence”.
- The use of abbreviation AdV species with the number of type like HAdV-C5 is not exactly appropriate, but widespread, so it is okay. But once it was misspelled (Methods/Assembly, 2 ^nd^ paragraph: “HAdVA-12” instead of “HAdV-A12”). Also here in the whole paragraph, while in abbreviation the first letter is indeed in capital like “HAdV-A”, in writing the names of viruses should be written by lower case like “human adenovirus A12” according to the recommendation of the ICTV (if the name is from a Latin genus/species name, like Odocoileus hemionus adenovirus, it is correct with capital).
- The name of fowl adenovirus in Methods/Assembly and in the legend of Figure 4 is incorrect (species D Fowl aviadenovirus 764). The correct name should be something like: “fowl adenovirus 8b strain 764 (FAdV-D)”.
- I would prefer the use of AdV type names (Arabic numbers) instead of species names (letters) on the phylogenetic trees, and GenBank numbers instead of RefSeq identification numbers (like starting with NC_...). These latter data won’t be updated, while the GenBank entries may be. Based on this, type data (including AC_... data) are more appropriate here.
- The examined AdVs are originated from rodent hosts. I missed very much other available AdVs with rodent origin on the phylogenetic trees, like squirrel AdV (KY427939), or murine adenovirus 2 (HM049560). If the data was too much for the calculation, I would skip rather MAdV-1 or MAdV-3. They are more similar to each other, while MAdV-2 is more different.
- Maybe this is a new practice, but for me, it was surprising and indistinctive, that the manuscript referred to itself.

I confirm that I have read this submission and believe that I have an appropriate level of expertise to confirm that it is of an acceptable scientific standard.

# Response

**Published:** 2020-03-06

**Authors:** Adriana Kajon

**Affiliations:** Lovelace Respiratory Research Institute, USA

We would like to thank Dr. Vidovsky for his comments and suggestions for improvement of our paper. Below are our responses to each individual comment/suggestion:

 This manuscript describes the isolation of two strains of guinea pig adenoviruses and their successful propagation on a newly developed and on three commercially available guinea pig (GP) cell lines. The manuscript is very relevant, as every successful, novel virus isolation is very important for adenovirology. The manuscript also discusses the partial phylogenetic comparison of adenoviral sequences with the examined AdV strains (full hexon gene and partial polymerase and penton base sequence parts). The Introduction is thorough enough and dense in a good meaning. It only deals with guinea pig AdVs, but all the known publications are well collected.

 -The Methods section is a bit too detailed and long compared to the other parts of the manuscript. Sometimes the process is already published somewhere, and the manuscript refers to it correctly but also details shortly. This is unnecessary in some cases in my opinion.

 Response:

*The detailed description of methods and reagents is an F1000 Research requirement.*

 -The process of the primary cell line production and its immortalization is very well-written. It is easy to follow the process and very interesting. The successful isolation and propagation of the two GPAdVs are a very nice job and well written. The pictures of CPEs on different cell lines represent the changes correctly and well visualize this part of the research. The surprising parts of the adenoviral PCR detection in the homogenates and infected cell cultures are that the original nasal scraping sample was PCR negative for AdV, while the cell culture infected by this material showed CPE and it was PCR positive. I would be curious of the opinion of the authors, how this can be possible, i.e., that the PCR reaction is not enough sensitive for detection, but the material is suitable for the usually less successful virus isolation.

 Response:

*We actually interpreted this result as a strong indicator that the original nasal scrapings sample contained infectious virus and that cell cultures were permissive for viral replication and amplification of the original input virus.*

 -It is a great result that the virus isolation attempts were successful with the GPAdV positive materials. It is more pity that the very interesting full genome sequence of these strains are not available. In the case of isolated viruses, it is only a small step by NGS. Because of the lack of full genome sequences of the examined AdVs, Figure 5 loses its relevancy. This makes some data incomparable in the figure. The phylogenetic calculations are very interesting, however, the one, based on the full hexon gene, seems to show a bit different topology than the other two. It is surprising, that among mastadenoviruses, not the rodent AdVs are the most ancient ones as usual, and they are not monophyletic with the GPAdVs. Summarizing, this is a well-written manuscript with very relevant and precious results.

 Response:

*Since the submission of this manuscript, we have been working on obtaining whole genome sequences for GPAdV and expect to generate publications in the near future reporting those results.*

 Minor notes:

 -There are a couple of not disturbing misspellings or inadvertent mistakes. The only one, which can be confusing is in the legend of Figure 5. The labeling is correct on the figure, but in the legend, both columns are labeled with blue instead of orange and blue.

 Response:

*We have corrected all spelling mistakes/typos in the body of the manuscript and figure legends.*

 Other mistakes:

 -Misspelling in the abstract/background: “variable” instead of “variable”. Twice, the word “resuspended” was misspelled to “resuspened” (Methods/Immortalization, 1 paragraph). Also, here in the last sentence the word “aliquoted” was misspelled to “alliquoted”. In most of the times, it was used correctly, but several times the “u” character was used instead of“µ” e.g.: twice in Methods/Immortalization, 2  paragraph. Also, here a space is missing in one of the cases, between the number and the unit (1ug), correctly (1 µg). The lack of space mistake between the amount and the unit occurs several times in the manuscript. I won't detail all of them here. Please check them. Also, a dot is unnecessary in the middle of a sentence of this paragraph, after a catalog nr: (catalog # MF10096).

 Response:

*All of these corrections were incorporated into the revised version of the manuscript.*

 -A comma should be used in the listing instead of “and” at Methods/GPTEC-T cell line authentication, after (DSMZ ACC 057).

 Response:

*The comma was NOT inserted because cell line 104 C1 was NOT obtained from the DSMZ collection. The text was edited to read **“... HeLa (DSMZ ACC 057) and 293 (DSMZ ACC 305), and cell line 104C1 (ATCC CRL-1405) were used as negative controls”***

 -The shortening of fowl adenovirus D was used incorrectly. The letter “a” of adenovirus is in lower case font. It should be a capital “A”. Everywhere else it was used correctly. (Methods/Assembly, 1 paragraph: “FadV-D” was written instead of “FAdV-D”). Once, the word “adenovirus” was written also with a capital incorrectly in the case of cynomolgus adenovirus. A “3’” end is missing for primer GPhexFW3 in Table 1. The word “cytopathic” was misspelled to “cythopathyc” (legend of Figure 3.). The word “twenty” was misspelled to “twety” (legend of Figure 4.). Also, here the word “absence” was misspelled to “absense”. The use of abbreviation AdV species with the number of type like HAdV-C5 is not exactly appropriate, but widespread, so it is okay. But once it was misspelled (Methods/Assembly, 2 paragraph: “HAdVA-12” instead of “HAdV-A12”). Also here in the whole paragraph, while in abbreviation the first letter is indeed in capital like “HAdVA”, in writing the names of viruses should be written by lower case like “human adenovirus A12” according to the recommendation of the ICTV (if the name is from a Latin genus/species name, like Odocoileus hemionus adenovirus, it is correct with capital).

 The name of fowl adenovirus in Methods/Assembly and in the legend of Figure 4 is incorrect (species D Fowl aviadenovirus 764). The correct name should be something like: “fowl adenovirus 8b strain 764 (FAdV-D)”.

 Response:

*All of these corrections were incorporated into the revised version of the manuscript.*

 -I would prefer the use of AdV type names (Arabic numbers) instead of species names (letters) on the phylogenetic trees, and GenBank numbers instead of RefSeq identification numbers (like starting with NC_...). These latter data won’t be updated, while the GenBank entries may be. Based on this, type data (including AC_... data) are more appropriate here.

 Response:

*The original designations NC_034834 and NC_000942, were changed to KY306667 and AC_000012, respectively as requested in the Methods section and in revised Figures 4 and 5.*

 -The examined AdVs are originated from rodent hosts. I missed very much other available AdVs with rodent origin on the phylogenetic trees, like squirrel AdV (KY427939), or murine adenovirus 2 (HM049560). If the data was too much for the calculation, I would skip rather MAdV-1 or MAdV-3. They are more similar to each other, while MAdV-2 is more different.

 Response:

*We completely agree with Dr. Vidovszky’s comments. We have made recent observations of close similarities between the genomes of GPAdV and MAdV-2 so the revisions addressing his comments are extremely important and timely. We have therefore incorporated the sequences for MAdV-2 into our new analyses for figures 4 and 5 and have revised both figures, and underlying data file now designated “*GC Content values 030320.xls” *.*
